# Supplementary material for: Establishing the role of ATP for the function of the RIG-I innate immune sensor
Source: eLife. 2015 Sep 15;4:e09391. doi: 10.7554/eLife.09391 (PMC4622095; doi:10.7554/eLife.09391)
Supplement: Supplementary file 1. — Sequences of RNA ligands used in this study. All RNA ligands and the sites of pertinent modifications are shown with the corresponding shorthand designation for each RNA. DOI: http://dx.doi.org/10.7554/eLife.09391.021 [file elife09391s001.docx]

**Experimental Procedures – Supplementary Table 1**

**Table Legend**

Table S1: Sequences of RNA ligands used in this study. All RNA ligands and the sites of pertinent modifications are shown with the corresponding shorthand designation for each RNA. All sequences are displayed 5’ to 3’.

| **Supplementary Table 1: Sequences of RNA Ligands** | |
| --- | --- |
| ***Designation*** | ***Sequence*** |
| 5’OH10L | OH:GGACGUACGUUUCGACGUACGUCC |
| 5’ppp10L | ppp:GGACGUACGUUUCGACGUACGUCC |
| 5’OH10L-FAM | OH:GGACGUACGUUU-[6FAM]-CGACGUACGUCC |
| 5’ppp14L | ppp:GGAUCGAUCGAUCGUUCGCGAUCGAUCGAUCC |
| 5’ppp30L | ppp:GGAUCGAUCGAUCGAUCGGCAUCGAUCGGCUUCGGCCGAUCGAUGCCGAUCGAUCGAUCGAUCC |
| 5’ppp50L | ppp:GACUACACGAAAGCUCCAAUUGUAUGCCAGGGUACAUCAUGCUGCGCAUCUUCGGAUGCGCAGCAUGAUGUACCCUGGCAUACAAUUGGAGCUUUCGUGUAGUC |
| Dumbbell:Top | p:GAGGGUACGGUCUGGGCUUCAA |
| Dumbbell-Cy3:Top | p:GAGGGUACGGUCUGGGCUU-[Cy3]-CAA |
| Dumbbell:Bot | p:GAGGCCCAGACCGUACCUUCAA |
